# Behavioral and corticosterone responses to carbon dioxide exposure in reptiles

**DOI:** 10.1371/journal.pone.0240176

**Published:** 2020-10-06

**Authors:** Daniel J. D. Natusch, Patrick W. Aust, Syarifah Khadiejah, Hartini Ithnin, Ain Isa, Che Ku Zamzuri, Andre Ganswindt, Dale F. DeNardo

**Affiliations:** 1 Department of Biological Sciences, Macquarie University, North Ryde, NSW, Australia; 2 EPIC Biodiversity, Frogs Hollow, NSW, Australia; 3 Department of Zoology, University of Oxford, Oxford, United Kingdom; 4 Bushtick Environmental Services, Grantham, Lincolnshire, United Kingdom; 5 Department of Wildlife and National Parks, Peninsular Malaysia, Kuala Lumpur, Malaysia; 6 Endocrine Research Laboratory, Mammal Research Institute, Department of Zoology and Entomology, Faculty of Natural and Agricultural Sciences, University of Pretoria, Pretoria, South Africa; 7 Centre of Veterinary Wildlife Studies, Faculty of Veterinary Science, University of Pretoria, Pretoria, Onderstepoort, South Africa; 8 School of Life Sciences, Arizona State University, Tempe, Arizona, United States of America; University of Texas at Arlington, UNITED STATES

## Abstract

The use of carbon dioxide (CO_2_) exposure as a means of animal euthanasia has received considerable attention in mammals and birds but remains virtually untested in reptiles. We measured the behavioral responses of four squamate reptile species (*Homalopsis buccata*, *Malayopython reticulatus*, *Python bivitattus*, and *Varanus salvator*) to exposure to 99.5% CO_2_ for durations of 15, 30, or 90 minutes. We also examined alterations in plasma corticosterone levels of *M*. *reticulatus* and *V*. *salvator* before and after 15 minutes of CO_2_ exposure relative to control individuals. The four reptile taxa showed consistent behavioral responses to CO_2_ exposure characterized by gaping and minor movements. The time taken to lose responsiveness to stimuli and cessation of movements varied between 240–4260 seconds (4–71 minutes), with considerable intra- and inter-specific variation. Duration of CO_2_ exposure influenced the likelihood of recovery, which also varied among species (e.g., from 0–100% recovery after 30-min exposure). Plasma corticosterone concentrations increased after CO_2_ exposure in both *V*. *salvator* (18%) and *M*. *reticulatus* (14%), but only significantly in the former species. Based on our results, CO_2_ appears to be a mild stressor for reptiles, but the relatively minor responses to CO_2_ suggest it may not cause considerable distress or pain. However, our results are preliminary, and further testing is required to understand optimal CO_2_ delivery mechanisms and interspecific responses to CO_2_ exposure before endorsing this method for reptile euthanasia.

## Introduction

Ensuring the humane euthanasia of animals used by humans is critically important to fulfil our ethical obligation for compassion towards other species. In addition, a painless and distress-free death can, in some contexts, result in a higher quality meat product for human consumption [1]. In pursuit of these goals, methodologies, guidelines, and regulations for humane euthanasia have been developed and implemented for animal use ranging from meat production to scientific research [2].

However, a severe taxonomic bias currently exists. Although humane treatment protocols are well established for mammals and birds, the welfare needs of reptiles and the methodologies considered humane and acceptable for euthanasia, especially in instances where human consumption of part of the carcass occurs, remain in their infancy [2]. For example, debate continues about the appropriateness of hypothermia (freezing) as an euthanasia method [3–5], and humane killing methods for reptiles used in the meat and skin industries were only adopted by the World Organization for Animal Health (OIE) in 2019 [see 6, 7].

Chemical agents offer an effective and humane way to euthanize reptiles, but their usefulness is sometimes limited. Access and use restrictions, and situations where large numbers of animals are slaughtered for human consumption in short periods, often prohibit their use. With the possible exception of hypothermia, all recommended non-chemical methods of reptile euthanasia involve destruction of the brain (e.g., captive bolt, pithing). However, the effectiveness of brain destruction is vulnerable to operator error and may be impractical in situations where large numbers of animals need to be killed at one time.

Carbon dioxide (CO_2_) is widely used as a euthanizing agent in the livestock industry and for scientific research [2, 8–10]. The guidelines of the American Veterinary Medical Association cite 86 studies on the effectiveness and suitability of CO_2_ as a humane means of euthanasia for mammals and birds [2]. Mammalian and avian responses to CO_2_ exposure vary considerably by species, and are dependent on CO_2_ concentration and delivery method [2, 8–10]. Mice, rats, cats, dogs, pigs, rabbits, chickens, and turkeys lose consciousness after 20–120 seconds of CO_2_ exposure, but may require exposures of 5–50 minutes to ensure death [2, 9, 10]. Exposure to CO_2_ has been shown to increase plasma corticosterone levels in rats and dogs and results in mouth gaping in mice, rats, and chickens [2, 9]. Rats and mink will actively avoid CO_2_ exposure if given the opportunity, but goats and chickens will not (despite the latter gaping when exposed; [2, 8]).

The use of CO_2_ to euthanize reptiles has generally been discouraged by veterinary guidance, animals ethics committees, and by the OIE based on physiological considerations [2, 6, 11, 12]. The rationale implies that because reptiles have a variable metabolic rate and can potentially tolerate long periods without breathing or oxygen, they are vulnerable to the distressful effects of suffocation. However, to the best of our knowledge the argumentation against using CO_2_ to euthanize reptiles lacks empirical data and rests instead upon untested hypotheses and theoretical inference.

Here, we examine the efficacy of CO_2_ to humanely euthanize squamate reptiles (lizards and snakes). Specifically, we tested the potential value of CO_2_ in (1) creating a low-stress, temporary unconscious state to make physical methods of euthanasia safer and more efficient and (2) killing squamates outright. We used both behavioral responses and blood corticosterone concentrations (the primary glucocorticoid associated with stress in reptiles) to determine whether CO_2_ exposure provides a humane transition to unconsciousness and examined how duration of CO_2_ exposure influences the post-exposure duration of unconsciousness and likelihood of death.

## Materials and methods

### Study species and locations

Behavioral responses to CO_2_ exposure were examined in four species of reptile: reticulated pythons (*Malayopython reticulatus*); Burmese pythons (*Python bivittatus*); masked water snakes (*Homalopsis buccata*); and Asian water monitors (*Varanus salvator*). These species are semi-aquatic to varying degrees and wide-ranging in Southeast Asia. The two python species grow to be large (> 5 m), while masked water snakes are relatively small (< 1.2 m). Asian water monitors are the world’s second largest lizard, growing to 3 metres in length and weighing as much as 25 kg. In many instances, these species are commensal with humans and are regularly harvested and traded for their meat, skin, and medicinal value.

In May 2019, we examined responses to CO_2_ in these reptiles in Malaysia (2°14’N, 103°03’E) and Thailand (17°38’N, 100°07’E) at two commercial facilities producing meat for human consumption and skins for the exotic leather trade. In Malaysia, free-roaming *M*. *reticulatus* and *V*. *salvator* are legally collected from the wild by licensed hunters and brought to abattoirs for processing [13, 14]. Animals are kept alive at the facility for up to a week before being killed using a strong blow to the head followed by decapitation. No individual-based history was available for the animals used in our study, and animals were held according to standard commercial protocols (i.e., maintained individually in mesh bags with water provided intermittently). In Thailand, we examined specimens of *M*. *reticulatus*, *P*. *bivittatus*, and *H*. *buccata*. The two python species were captive-bred for commercial purposes following protocols described in Natusch and Lyons [15]. The *H*. *buccata* were wild-caught and temporarily held in large outdoor ponds with food provided. This research was undertaken with approval from the Animal Institutional Care and Use Committee of Arizona State University (protocol # 10-1689R).

### Experimental design—behavioral monitoring

To assess behavioral responses of reptiles to CO_2_ exposure, we placed study animals individually into 100 micron 375 mm x 500 mm clear plastic bags. Very large animals were double-bagged as a precaution. CO_2_ was supplied via 47 litre steel cylinders containing 99.5% CO_2_ and fitted with single-stage CO_2_ regulators. A 5 mm inside diameter CO_2_ supply hose was placed in the bag through the opening at the top, and the bag was sealed with an elastic band to limit but not eliminate the escape of gas. Bags were gently compressed around the body of the animal prior to CO_2_ admission to minimize residual air pockets. This design enabled CO_2_ to rapidly displace the limited amount of air present in the bag and thus minimized gas equilibration time [16]. By using plastic bags instead of a rigid container, we were able to closely evaluate the animal during its exposure to CO_2_ (e.g., examine the animal’s righting response and its response to touch stimulation). CO_2_ flow was set to rapidly replace any existing air and then reduced to maintain positive CO_2_ pressure in the bag. For the longer exposure times, once the animal was unconscious, the flow of CO_2_ was stopped and the bagged was completely sealed. The process was similar for water monitors except that the bag was secured over their head rather than placing the entire body inside the bag (to minimize damage to the plastic bag by the lizard’s claws). We prevented monitors from perforating the bag during movements by gently placing a hand around the animal’s neck and preventing the forelimbs from contacting the bag. For some individuals this was not necessary and did not prevent observation of general body movements in response to CO_2_ exposure. For all individuals, the response of the animal to CO_2_ exposure was recorded via direct visual examination until the animal was removed from the bag after the duration of CO_2_ exposure dictated by its assigned treatment group.

For each animal, we recorded signs of consciousness and all behavioral responses to CO_2_, including movement, tongue flicking, and gaping. The animal’s behavior and body movements at the time of removal were recorded, as were changes in behavior over time and the eventual outcome (i.e., recovery or confirmed death). It was difficult to determine consciousness in many specimens. Although several individuals continued to respond to deep-touch stimuli (e.g., a deep pinch of the tail), a lack of righting reflex (failure to turnover when placed upside down), corneal reflex in lizards, and cessation of breathing, strongly indicated that individuals were unconscious despite exhibiting a muscular response to deep stimuli. Animals that reached a state indicative of imminent recovery of consciousness (i.e., voluntary movement often associated with tongue flicking) were euthanized using standard commercial practices (i.e., forceful blunt trauma to the dorsal surface of the head at the location of the brain case). Animals were deemed dead if no heartbeat and/or movements were detected (visually or via palpation) or by a lack of response to all stimuli (most notably a deep tail pinch) for up to one hour after removal from CO_2_ exposure.

To test the effect of CO_2_ exposure duration on reptile responses, we first conducted a preliminary assessment using different exposure durations on five *M*. *reticulatus* (30 min, 60 min, 90 min, 120 min, or 180 min; n = 1 per duration). Based on related observations, we selected three CO_2_ exposure durations (15 min, 30 min, and 90 min) for the primary study. We used the results from the reticulated pythons to select exposure durations for the other species. As our results from *M*. *reticulatus* showed that 15 min was an insufficient duration, we began studies of other species with the 30 min exposure duration to minimise the number of animals used and to streamline efforts. If all specimens of the species failed to recover at this exposure duration, we assumed longer durations would achieve the same result, so did not conduct longer duration trials. This was not true for *H*. *buccata* for which we did not complete the 90 min exposure treatment due to specimen availability and logistic constraints. We measured snout-vent length (SVL; using a steel tape measure) and body mass (using a digital scale) of each specimen while unconscious or dead, and then determined sex via direct inspection of the gonads upon dissection. Sample sizes for each species and their CO_2_ exposure times are presented in Table 1. Air temperature was recorded to confirm constant temperatures throughout the course of study.

**Table 1:** Means, standard errors and ranges for snout-vent length (SVL) and body mass for reptile specimens used to examine behavioral responses to CO_2_ exposure.

Species	Sex	N	SVL (cm)		Mass (g)		N per exposure duration		
			Mean	Range	Mean	Range	15 min	30 min	90 min
**Thailand**									
*Malayopython reticulatus*	M	1	273	-	8200	-	0	1	0
	F	3	265.3 ± 8.9	255–283	7200 ± 1790	4200–10400	0	3	0
*Python bivittatus*	M	18	241.5 ± 2.7	220–263	6941 ± 545	3900–11800	0	9	9
*Homalopsis buccata*	M	11	104 ± 2.2	93–116	686 ± 36	530–850	0	8	0
**Malaysia**									
*Malayopython reticulatus*	M	12	272.8 ± 8.6	238–331	7335 ± 728	4550–13450	3	4	4
	F	14	297.4 ±8.3	255–374	7878 ± 608	4050–12850	5	6	4
*Varanus salvator*	M	5	63 ± 3.3	50–68	4990 ± 708	2250–6350	0	5	0
	F	5	59 ± 3.8	52–71	4000 ± 714	2550–6000	0	5	0

### Experimental design–sample collection for hormone monitoring

We measured the effect of the CO_2_ euthanasia process on circulating corticosterone by collecting blood from seven *M*. *reticulatus* and seven *V*. *salvator* before and after CO_2_ exposure. Specimens were brought to the National Wildlife Forensic Laboratory, Department of Wildlife and National Parks Peninsular Malaysia. Sexes and body sizes are reported in Table 2. Each animal was kept individually within a mesh bag and secured within a plastic crate at ambient temperature for two days before trials began. We collected 2 ml of blood from each individual within 90 seconds of removal from the mesh bag using a 22 gauge needle and 5 ml syringe inserted into the caudal vein at the base of the tail. The blood sample was then placed in a tube containing lithium heparin (Vacuette #454084, Greiner Bio-One, Kremsmünster, Austria). After blood collection, the same specimens were immediately exposed to CO_2_. A second blood sample was collected from the same specimen after 15 minutes of CO_2_ exposure when the animal was unconscious. We did this by amputating the lower third of the tail and collecting the blood directly into a heparinized tube. The animal was then immediately euthanized following standard methods as described above. Blood samples were placed on ice until centrifugation to separate the plasma. We stored the isolated plasma samples at -20°C until they were assayed. As confinement in the mesh bag may in itself result in elevated levels of corticosterone, we collected blood samples from several ‘control’ animals for comparison. The control water monitors (n = 3) were freshly killed wild animals harvested during a government sanctioned control program in Ladang Eng Tai, Malaysia (4°57'N 100°27'E). Animals were harvested using a 12-gauge shotgun at close range, with head shots resulting in near-instantaneous death. We collected blood from the severed tail of each animal within 90 seconds using the same method described above. Control reticulated python (n = 4) samples were obtained from captive-bred animals at a commercial reptile breeding facility outside Kuala Lumpur, Malaysia (2°56'N 101°53'E). The farm breeds high-value pythons for the pet trade, and general husbandry and welfare standards are high. Animals were selected based on size and relative docility (i.e., ease of handling), and blood samples were collected from the caudal vein within 90 seconds of removal from their enclosures using the same method described above. We recorded temperatures (27–30°C) and kept all animals at approximately the same temperature both before and after exposure to CO_2_. This was not possible for control specimens sampled in the wild, but plasma corticosterone levels are not highly sensitive to body temperature in reptiles [17]. We obtained all blood samples over several hours on the same day to avoid diel and seasonal variation in plasma hormone levels.

**Table 2:** Means, standard errors and ranges for snout-vent length (SVL) and body mass for reptile specimens used to examine plasma corticosterone responses to CO_2_ exposure.

Species	Treatment	Sex	N	SVL (cm)		*Mass (g)*	
				Mean	*Range*	Mean	Range
*Malayopython reticulatus*	CO_2_	M	3	246 ± 5.6	235–255	4720 ± 204	4400–5100
		F	4	253.5 ± 4.6	240–260	5280 ± 225	4720–5800
	Control	M	2	295 ± 55	240–350	8500 ± 3500	5000–12000
		F	2	375 ± 25	350–400	35000 ± 0	35000
*Varanus salvator*	CO_2_	M	2	53.7 ± 1.8	51–57	2830 ± 233	2600–3300
		F	5	56.2 ± 2.9	47–63	2900 ± 370	1500–3750
	Control	M	2	79 ± 10	69–89	7850 ± 2350	5500–10200
		F	1	69	-	6500	-

### Hormone analysis

Immunoreactive plasma corticosterone concentrations were determined via an enzyme-linked immunosorbent assay (ELISA; ADI-900-097, Enzo Life Sciences, Farmingdale, NY) following the manufacturer’s instructions. This kit has been used in previous studies assessing plasma corticosterone concentrations in a variety of animal species, including alligators [18], birds [19], lizards [20] and turtles [21], but had not been previously documented for pythons or monitor lizards. Based on results from other species, we used a dilution ratio of 40:1. All samples were run in duplicate format on a single assay plate. Results confirmed an average difference between duplicates of less than 1.8% (mean: 1.73 ± 1.18%), and duplicate means were thus used in the analysis.

### Data analysis

Our behavioral analysis measured the binary dependent variable of whether reptiles recovered after CO_2_ exposure or not. This metric was evaluated after different CO_2_ exposure durations for each species. For our corticosterone study we used a paired sample t-test to test for significant differences in plasma corticosterone concentrations before and after CO_2_ exposure. We used a one-way analysis of variance to test for differences in corticosterone level between the control animals and the pre-CO_2_ exposure samples from the study animals. Data were ln-transformed where needed to meet the normality and homogeneity of variance assumptions required for our parametric tests. All analyses were conducted in JMP Pro 14 (SAS Institute, Cary, NC).

## Results

### Behavioral observations

#### Reticulated pythons (*Malayopython reticulatus*)

**Fig 1:**
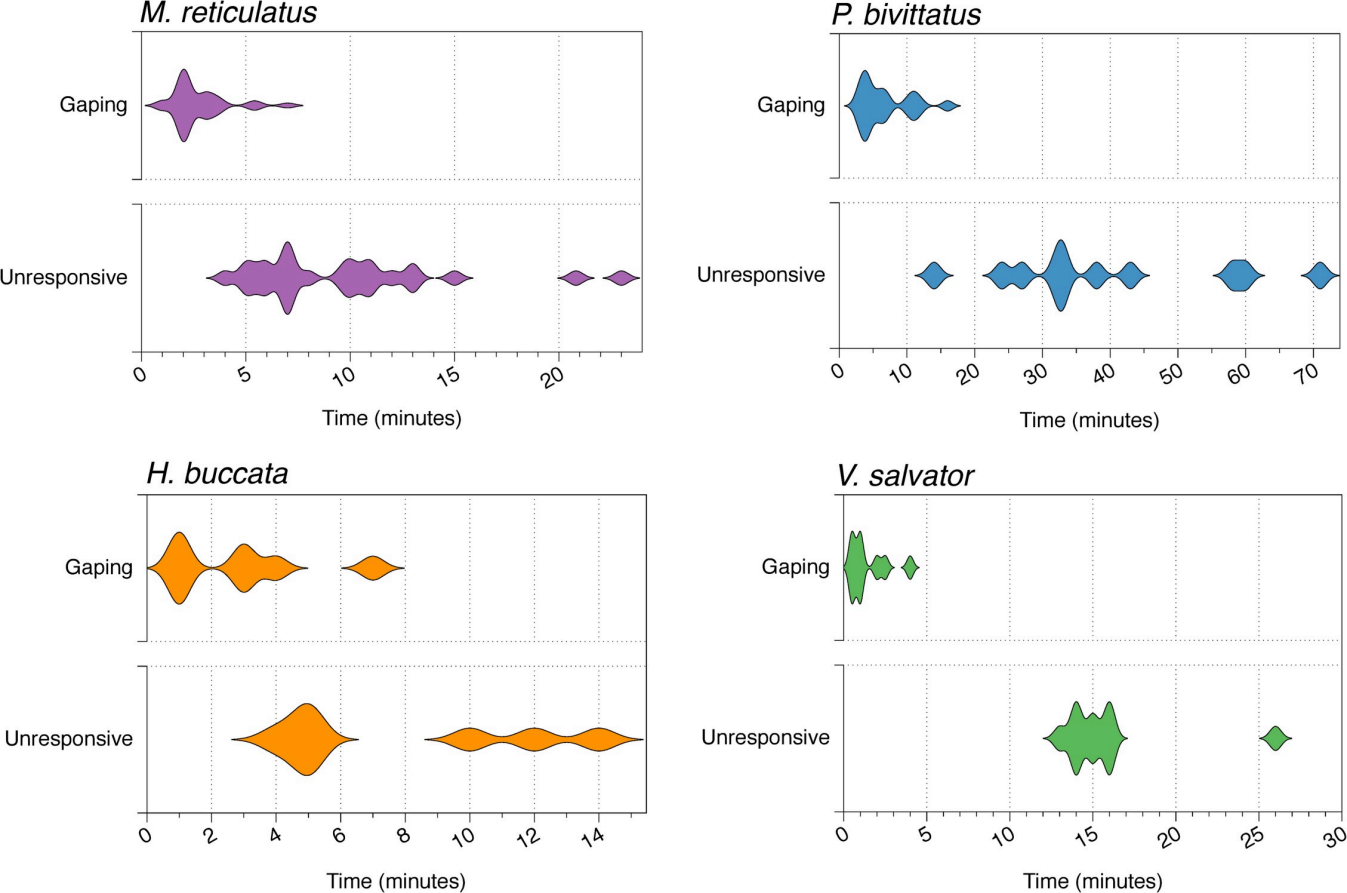
Variation in timing (in minutes) of key behavioural changes in (a) *Malayopython reticulatus*, (b) *Python bivittatus*, (c) *Homalopsis buccata*, and (d) *Varanus salvator* subject to carbon dioxide (CO_2_) exposure. Gaping: the time at which the mouth of the specimen opened. Unresponsive: the time the specimen had ceased movement and became unresponsive to stimuli. Thicker parts of the violin plots represent CO_2_ exposure times where the behaviour was most often observed. Note the different time scales represented on the x-axes of each panel.

After exposure to CO_2_, reticulated pythons remained still for 60–300 secs (1–5 mins) before tongue flicking and gaping (Fig 1). These responses eventually proceeded to slow and controlled whole-body movements; at this time snakes were responsive to touch through the bag. It was difficult to determine the point at which snakes lost full consciousness. However, we suspect that snakes lost consciousness, but continued to undergo unconscious movements including a response to touch stimuli. Between 240–1380 secs (4–23 mins) after CO_2_ exposure the snakes ceased all movements and lost responsiveness to stimuli (Fig 1). After the cessation of movement, but sometimes before, 18 of the 30 snakes exhibited mild muscle twitching of parts of their body. This twitching was unique to the reticulated pythons.

**Fig 2:**
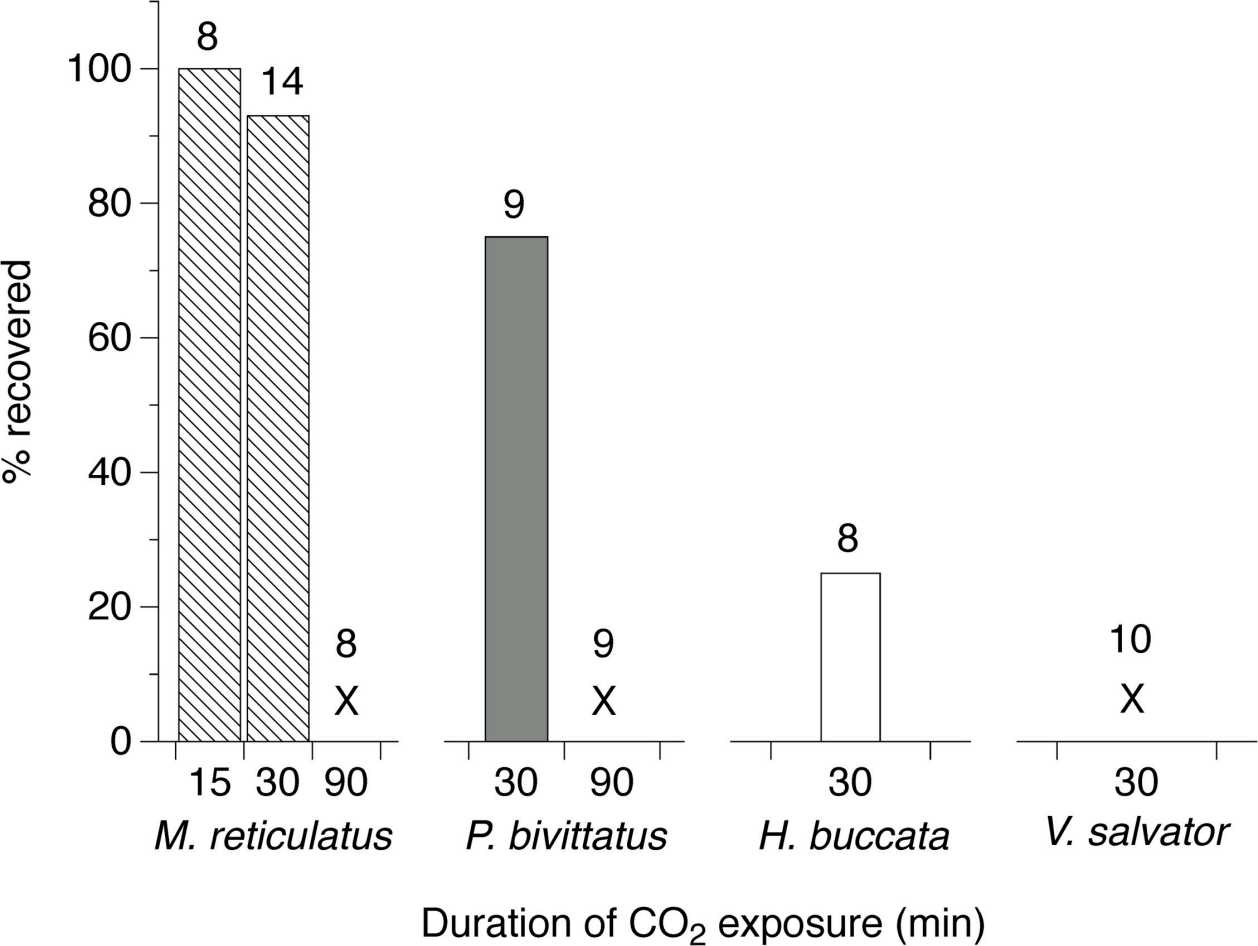
Percentage of *Malayopython reticulatus*, *Python bivittatus*, *Homalopsis buccata*, and *Varanus salvator* that recovered from different durations of CO_2_ exposure. X denotes treatments where no individuals recovered from CO_2_ exposure. Sample sizes appear above each column.

All Malaysian reticulated pythons that were exposed to CO_2_ for 15 and 30 min eventually recovered (Fig 2). At the time of removal from the bag, none of these snakes had voluntary movements, but 7 of 8 snakes in the 15-min exposure group and 1 of 10 snakes in the 30-min group responded to a deep tail pinch with local movement. First voluntary movements occurred 4.9 ± 0.9 (mean ± SE) and 23.8 ± 4.7 min after removal from CO_2_ for the 15 min and 30-min exposure groups, respectively. In contrast, all reticulated pythons exposed to 90-min of CO_2_ did not recover, never having any reflex or voluntary movements (Fig 2). Reticulated pythons tested in Thailand that were exposed to CO_2_ for 30 min responded similarly to those in Malaysia, but one of the four snakes did not recover and, for those that did, recovery took 13.7 ± 3.7 min (42% faster than the 30-min exposure snakes in Malaysia).

#### Burmese pythons (*Python bivittatus*)

Burmese pythons showed similar behavioral responses to reticulated pythons, but took slighter longer to gape and lose responsiveness to stimuli (Fig 1). Burmese pythons also did not undergo muscle twitching and late-stage non-responsive (likely unconscious) movements were greater. All 8 snakes in the 30-min group responded to a deep tail pinch upon removal from the CO_2_, while none of the 90-min snakes responded. Two of the 8 snakes exposed to CO_2_ for 30 min and all of the snakes exposed to CO_2_ for 90 min did not recover (Fig 2). For the six 30-min snakes that did recover, it took 17.4 ± 2.5 min until they showed their first voluntary movements.

#### Masked water snakes (*Homalopsis buccata*)

The water snakes exposed to CO_2_ for 30 min showed behavioral responses that were very similar to those of the Burmese pythons, with no twitching but a considerable amount of unconscious movements. Mean time of first gape was about 120 secs (range: 60–420 secs, 1–7 min) and complete loss of consciousness was 300–840 secs (5–14 mins) after the onset of exposure (Fig 1). While all eight water snakes had a tail pinch reflex upon removal from the CO_2_, only two of the eight snakes recovered after 10 and 20 min, respectively.

#### Water monitors (*Varanus salvator*)

The water monitors showed the least behavioral response to exposure to CO_2_. The lizards exhibited no tongue flicking and no muscle twitching during the 30 min exposure. All monitors gaped within 240 secs (4 mins) of the onset of CO_2_ exposure (Fig 1) Both conscious and unconscious movements were limited in number and intensity with the last detected movements occurring 930 ± 66 secs (range: 720–1560 seconds) after the onset of exposure (Fig 1). All monitors lacked a tail pinch reflex when removed from the CO_2_, and they all failed to recover (Fig 2).

### Plasma corticosterone concentrations

**Fig 3:**
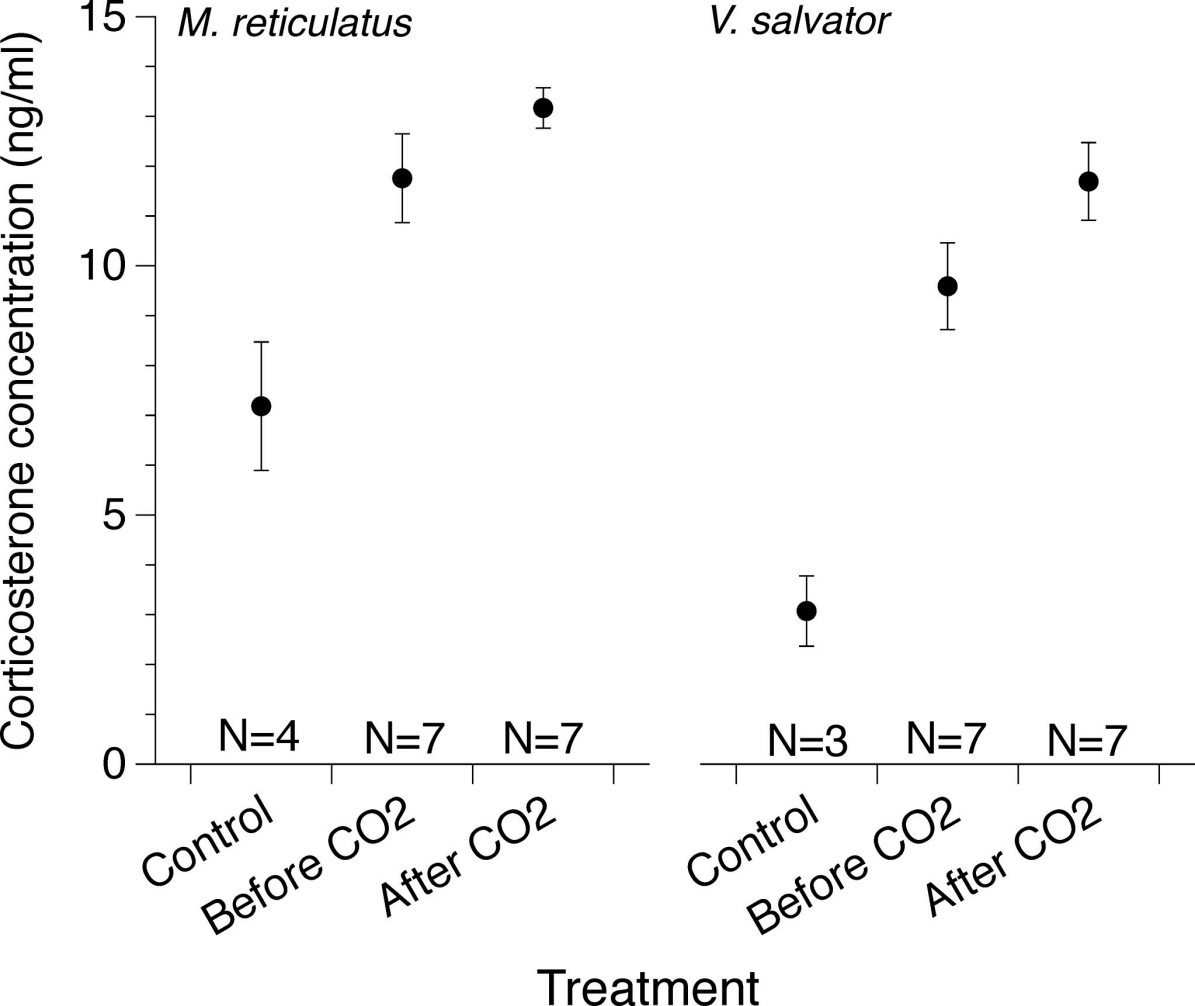
Mean plasma corticosterone concentrations (ng/ml) before and after 15 minutes of CO_2_ exposure and in control specimens (free-ranging or farmed; see text) of (a) *Malayopython reticulatus* and (b) *Varanus salvator*. Differences between corticosterone concentrations before and after CO_2_ exposure were not statistically significant for *M*. *reticulatus*, but were for *V*. *salvator*. Corticosterone concentrations between control specimens not subject to capture and handling are significantly lower than those captured from the wild for trade (although sample sizes were low; see text for details). Sample sizes for each group are reported directly above the x-axis.

Corticosterone concentrations for the animals that did not go through the capture and confinement associated with the trade prior to killing (i.e., ‘controls’) were significantly lower than those of the CO_2_-euthanized animals prior to CO_2_ exposure (pythons: 7.2 ± 1.3 ng/ml; F_1,10_ = 9.01, P = 0.015; monitors: 3.1 ± 0.7 ng/ml; F_1,10_ = 24.4, P < 0.001; Fig 3). Reticulated python plasma corticosterone concentrations increased by 14% after CO_2_ exposure, (t_0_ = 11.8 ± 0.9 ng/ml vs t_15_ = 13.2 ± 0.4 ng/ml). However, this increasing trend was not statistically significant (matched pairs t-test: t_6_ = 2.23, P = 0.065; Fig 3). In contrast, CO_2_ exposure significantly increased plasma corticosterone concentrations in water monitors (by 18%; t_0_ = 9.6 ± 0.9 ng/ml; t_15_ = 11.7 ± 0.8 ng/ml; t_6_ = 5.03, P = 0.02; Fig 3). Individual immunoreactive plasma corticosterone concentrations before and after CO_2_ exposure were significantly correlated (pythons: n = 7; *r*^2^ = 0.61; P = 0.037; lizards: n = 8; *r*^2^ = 0.77; P = 0.009).

## Discussion

Although available euthanasia methods for commercial reptile processing (e.g., brain destruction) are humane, they can be vulnerable to operator error, are aesthetically displeasing, and are inefficient for rapidly processing numerous individuals. Our study provides initial results supporting the potential for carbon dioxide asphyxiation as an effective option for euthanizing reptiles in a variety of settings. Exposure to CO_2_ was effective for creating a temporary unconscious state at all exposure durations that was sufficient to safely and humanely employ a physical method of euthanasia. Longer but still logistically practical exposures to CO_2_ were able to kill reptiles.

The different taxa in our study varied subtly in their responses to CO_2_ exposure, both while conscious and after losing consciousness. For example, despite the similar body size of the two python species, the CO_2_ exposure duration required to induce unconsciousness in *P*. *bivittatus* was greater than *M*. *reticulatus* (Fig 1). The only lizard species in our study was rapidly rendered unconscious and did not recover from CO_2_ exposure durations that were unable to kill most of the snakes (Fig 2). Taxonomic differences and variation in metabolic rates may both be responsible for this difference [22–24]. The species we studied also differed in the effects that a given duration of CO_2_ exposure had once the animal was removed from CO_2_, including the extent of involuntary/reflex muscle activity and the likelihood of death. Unfortunately, we did not have a sufficient sample size to examine sexual differences in species’ responses to CO_2_ exposure. Plausibly, CO_2_ may affect males and females differently, especially in those species with strong sexual dimorphism. Related to this, our study was undertaken on several of the world’s largest reptiles, all of which are semi-aquatic and can remain submerged under water for considerable periods. Application of CO_2_ exposure to the myriad of smaller-bodied reptiles, and to strictly terrestrial species, may yield different results.

We made the assumption that because the density of CO_2_ is greater than air, complete (100%) CO_2_ saturation would occur as air was expelled from the small opening positioned at the top of the bag [25]. However, we did not directly measure the concentration of CO_2_ within the bag and whether the concentration was homogenous. Layering of CO_2_ could enable specimens to avoid exposure [2]. The variation in responses to CO_2_ exposure in our study may be related to minor but functionally significant difference in CO_2_ distribution [see 26]. In order to more broadly apply CO_2_ as a euthanasia method in reptiles, there needs to be a better understanding of interspecific difference among taxa as well as a delivery system with established displacement parameters and sufficient holding capacity.

Regardless of species, our behavioral observations suggest the reptiles used in our study do not suffer significant distress from CO_2_ exposure. Although our observational assessments were subjective, the body movements made by conscious reptiles were minor and appeared considerably less vigorous than the escape behavior displayed by these same animals when first removed from their holding bags. In the case of *V*. *salvator*, some specimens went unconscious without showing any signs of movement. Nevertheless, it is challenging to accurately determine if reptiles are indeed dead, let alone feeling pain, based solely on behavioral responses [27, 28]. For example, an active heartbeat, involuntary movements, and response to touch stimuli can continue for hours after complete destruction, pithing, and removal of the brain [Natusch unpubl. data 2020, 2]. Similarly, our data on the time reptiles take to lose responsiveness are difficult to interpret. It was often unknown if specimens were consciously responsive, or unconscious and merely exhibiting involuntary muscular reflex. Importantly, the difficulty of assuring death, and the high but less than 100% effectiveness at killing at some CO_2_ exposure durations, may warrant the use of a secondary method to ensure death as is commonly used for chemical-induced euthanasia of research animals [see 2].

The most consistent behavioral response to CO_2_ exposure was the non-violent gaping displayed by most (90%) individuals. Gaping is common in mammals and birds subject to CO_2_ exposure, and in birds does not appear to be a sign of distress when exposed to CO_2_ [29]. It is unknown whether gaping is a sign of significant distress in reptiles. Gaping occurred within 30 seconds to 16 minutes of initiating CO_2_ exposure and the timing varied among taxa (Fig 1). The short duration between initial exposure and gaping, and then unconsciousness, suggests that suffocation may not be the cause of death in reptiles exposed to CO_2_. All species used in our study are semi-aquatic, and capable of spending significant time underwater (>20 minutes), suggesting another physiological response is taking place. Despite the lack of behavioral indicators for stress and pain, reptiles take considerably longer to lose consciousness than mammals and birds [30–32]. Some consider a gentle death that takes longer is preferable to a rapid but more distressing death [26, 33]. In the context of CO_2_ and reptiles, further research is needed.

Our additional approach to investigate the impact of CO_2_ exposure in our study species, by monitoring plasma corticosterone concentrations, also suggests that reptiles experience relatively minor distress from CO_2_ exposure. Comparison to our control (wild or farmed) specimens suggests the relative increase in stress involved in restraint and transportation of specimens to the laboratory was greater than the distress induced by CO_2_ exposure [2, 34]. Brown tree snakes (*Boiga irregularis*) and red-sided garter snakes (*Thamnophis sirtalis*) captured and placed in bags for 2–4 hours increased plasma corticosterone levels by 280–1200% [35, 36], but resulted in no appreciable increase in corticosterone concentrations in bearded dragons (*Pogona barbata)* [37]. Several studies reveal a lack of adverse impacts of corticosterone increase on survival, feeding behavior, and reproduction [38–40]. Other studies document invasive procedures (e.g., toe clipping, microchipping) inducing smaller corticosterone increases than did natural stresses experienced in the wild [27]. The relatively small increases in plasma corticosterone concentrations observed in pythons (14%) and lizards (18%) in our study may suggest that the functional relevance (distress or pain) of CO_2_ exposure-induced increases in corticosterone may be negligible. It is possible that the small increases in corticosterone levels we observed were related mostly to the stress caused by restraining and collecting an initial (T_0_) blood sample from each specimen, rather than by exposure to the CO_2_ itself. Alternatively, a post-CO_2_ exposure increase in corticosterone may have been suppressed because the recent capture, confinement, and handling had already maximized the hypothalamic-pituitary-adrenal (HPA) axis response.

Intriguingly, exposure to CO_2_ may have additional benefits beyond the possibility of a painless death. After death, animals can have spinal cord induced muscle activity, and this can last for an extended duration in reptiles due to their tissue’s high tolerance of hypoxia. This phenomenon can lead to the impression that the animals is still alive [2], and thus has been capitalized on by activists who oppose the consumption of animals, claiming they are being processed while still alive. In addition to being aesthetically displeasing, continued muscle movements after death force staff in commercial facilities to delay the harvesting of tissues for up to two hours after death [41]. When killed via CO_2_ exposure, we recorded no involuntary muscle movements after the presumed point of death, including during the processing of the reptiles. The physiological cause of this lack of muscle tone is unknown but, given its functional and cosmetic advantages, warrants further investigation.

In conclusion, our study presents some of the first results on the effects of CO_2_ exposure in reptiles. We stress that our results are preliminary and therefore are reluctant to recommend CO_2_ as a humane method of reptile euthanasia at this time. Despite our results being generally positive, we identified some interspecific differences and methodological variables that may influence the effectiveness of CO_2_ exposure. Future studies could usefully disentangle the influence of these variables and employ alternative methods for assessing stress, pain, and death in reptiles (e.g., electroencephalography).

## Supporting information

S1 Data CO analyses. (XLSX)
